# Potential oncogenic role of occult hepatitis B virus pre-S mutations: Activation of Akt/mTOR/Cyclin D1 signaling drives cell cycle dysregulation and proliferation in hepatocellular carcinogenesis

**DOI:** 10.1016/j.gendis.2025.101919

**Published:** 2025-11-06

**Authors:** Huizhen Sun, Ling Mei, Shi Song, Qian Su, Ying Yan, Huimin Ji, Jie Ma, Le Chang, Lunan Wang

**Affiliations:** a National Center for Clinical Laboratories, Beijing Hospital, National Center of Gerontology, Institute of Geriatric Medicine, Chinese Academy of Medical Sciences, Beijing 100730, China; b Beijing Engineering Research Center of Laboratory Medicine, Beijing 100730, China; c National Center for Clinical Laboratories, Peking Union Medical College, Chinese Academy of Medical Sciences, Beijing 100730, China

**Keywords:** Akt/mTOR signaling, Cell cycle, HBV pre-S mutations, Occult hepatitis B virus infection, Proliferation

## Abstract

Hepatitis B virus (HBV) infection remains a severe global public health challenge, with hepatocellular carcinoma being a primary cause of HBV-related mortality. Occult HBV infection (OBI) represents a distinct type of HBV infection that has been increasingly linked to hepatocellular carcinoma development, yet the precise molecular mechanisms underlying this association remain poorly elucidated. Although HBV pre-S deletion mutations have been shown to enhance cell proliferation and contribute to hepatocarcinogenesis, the biological functions of other types of pre-S mutations, particularly point mutations, are still mostly unexplored. In our prior studies, we identified several high-frequency pre-S point mutations from OBI blood donors. Within this research, we systematically explored the effects of these OBI-associated pre-S mutations on host cell proliferation and assessed their potential oncogenic properties. Cell proliferation assays revealed that several pre-S mutations significantly enhanced the proliferative capacity of host cells. Mechanistically, five pre-S mutations (E39K, D44N, N98T, H128R, and I161T) activated the Akt/mTOR signaling cascade, up-regulated Cyclin D1 expression, and induced G1-to-S phase cell cycle progression. Further analyses suggested that the large HBV surface protein (LHBs) likely acts as the key mediator linking pre-S mutations to signaling activation and cellular proliferation. These findings provide novel mechanistic understandings of the oncogenic potential of pre-S point mutations in hepatocarcinogenesis and may facilitate the identification of high-risk individuals within OBI populations as well as the development of treatment strategies for hepatocellular carcinoma linked to HBV.

## Introduction

Hepatitis B virus (HBV) infection remains a considerable health challenge all over the world, serving as a key driver of chronic liver disease. In 2022 alone, it was responsible for approximately 1.1 million deaths, primarily attributed to cirrhosis and hepatocellular carcinoma (HCC).^1^^,^^2^ Occult HBV infection (OBI) represents a unique entity within the spectrum of HBV infections, referring to a condition in which hepatitis B surface antigen (HBsAg) is undetectable in the blood by current available approaches, while replication-competent HBV DNA persists in the liver and/or low levels of viral DNA can be detected in the blood (typically < 200 IU/mL).^3^ The global prevalence of OBI differs and shows a close link to the regional endemicity of HBV. The reported prevalence among blood donors ranges from approximately 0.06%–0.98%.^4^ Due to its concealed nature, OBI is often undiagnosed and may hamper accurate evaluation of antiviral treatment efficacy in clinical settings. Accumulating evidence suggested that OBI is closely associated with progressive liver disease and HCC. About 70% of cryptogenic HCC cases have been found to be OBI positive. Moreover, OBI was confirmed as an independent risk factor for cryptogenic cirrhosis progression to HCC.^5^, ^6^, ^7^, ^8^, ^9^ However, the precise molecular mechanisms by which OBI contributes to hepatocarcinogenesis remain unclear.

As a vital component of the HBV genome, the pre-S region, together with the downstream S region, encodes three viral surface proteins—small (SHBs), middle (MHBs), and large (LHBs). Notably, the pre-S region exhibits high genetic variability.^10^ Mutations in this region, particularly deletions, can promote hepatocyte proliferation via various oncogenic signaling pathways, thereby contributing to HCC development.^11^, ^12^, ^13^ In our previous work, we identified several high-frequency pre-S point mutations (amino acid substitutions) from a cohort of approximately 300 OBI blood donors. Among them, some mutations were found to impair HBsAg expression and contribute to the development of OBI.^14^ Nonetheless, studies investigating the effects of these pre-S point mutations on host cell proliferation, as well as their roles and underlying mechanisms in hepatocarcinogenesis, are still lacking. Consequently, the pathogenic significance and mechanistic roles of these high-frequency pre-S point mutations in OBI remain poorly understood, thereby posing challenges for accurate risk assessment in the large OBI population.

In this study, we focused on a set of previously identified OBI-associated high-frequency point mutations in the pre-S region, aiming to assess their impact on cellular proliferation and elucidate the underlying molecular mechanisms. This work may yield novel insights into the functional relevance of these mutations in hepatocarcinogenesis and may inform both risk stratification among individuals with OBI and the discovery of potential therapeutic targets in HBV-associated HCC.

## Methods and materials

### Construction of plasmids

We previously constructed a wild-type (WT) plasmid that contains the full-length HBV genome (1.3 × genotype B4) and subsequently constructed 20 pre-S mutant plasmids (MUT) based on the WT plasmid.^16^ The pcDNA 3.1-LHBs plasmid was constructed as follows: the pre-S/S region (nt 2848-3125/1-835) and the posttranscriptional regulatory element (PRE, nt 970-1770) of full-length HBV plasmids were inserted into the KpnI-XhoI sites of pcDNA 3.1 vector, which allowed highly efficient HBsAg expression.^15^ The initiating codons of MHBs and SHBs were sequentially mutated to ACG using site-directed mutagenesis, thereby abolishing the expression of MHBs and SHBs and ensuring the exclusive expression of LHBs. Sanger sequencing was adopted to verify the successful construction of all the plasmids.

### Cell culture and transfection

Huh-7 cells were maintained in DMEM (Gibco, New York, New Jersey, USA) supplemented with 10% fetal bovine serum (Gibco, New Zealand) and 1% penicillin-streptomycin (Gibco, New York, New Jersey, USA), under conditions of 37 °C and 5% CO_2_. Huh-7 cells were plated into 6-well plates 24 h before transfection. The WT/MUT HBV plasmids or pcDNA 3.1-LHBs plasmid were transfected into Huh-7 cells via the Lipofectamine™ 3000 Reagent (Invitrogen, Carlsbad, California, USA).

### Cell proliferation assay

Cells for the proliferation assay were collected 48 h after transfection and reseeded into 96-well plates with a seeding density of 5000 cells per well. CCK reagent (TransGen Biotech, Beijing, China) was introduced into 96-well plates following the manufacturer's recommended time and incubated at 37 °C for 2 h. The supernatant was then moved to a clean 96-well plate, and a microplate reader (Autobio Diagnostics Co., Ltd., Zhengzhou, China) was used to measure absorbance at 450 nm. All small-molecule inhibitors used in the cell proliferation assays were obtained from MCE (MedChemExpress LLC, Monmouth Junction, New Jersey, USA).

### Western blotting analysis

Cells that had undergone transfection were collected and lysed in NP-40 lysis buffer (Solarbio, Beijing, China) supplemented with 1% PMSF (Abcam, Cambridge, UK). The total proteins were separated through SDS-PAGE, followed by their transfer onto a PVDF membrane. (Merck Millipore, Darmstadt, Germany). Then, the membrane was blocked with 5% nonfat dry milk in TBST buffer, followed by incubation with primary antibodies and HRP-linked secondary antibodies. Bands were detected by ECL chemiluminescent reagent (NCM, Suzhou, China). The antibodies employed in this research included anti-mTOR (#2983, 1:1000, Cell Signaling Tech., Danvers, Massachusetts, USA), anti-Phospho-mTOR (ab109268, 1:1000, Abcam, Cambridge, UK), anti-Phospho-Akt (#4060, 1:1000, Cell Signaling Tech., Danvers, Massachusetts, USA), anti-Akt (#4691, 1:1000, Cell Signaling Tech., Danvers, Massachusetts, USA), anti-Phospho-p70 S6 Kinase (#9234, 1:1000, Cell Signaling Tech., Danvers, Massachusetts, USA), anti-p70 S6 kinase (sc-8418, 1:500, Santa Cruz Biotechnology, Dallas, Texas, USA), anti-Cyclin D1 (#2978, 1:1000, Cell Signaling Tech., Danvers, Massachusetts, USA), anti-Phospho-Rb (#85116, 1:1000, Cell Signaling Tech., Danvers, Massachusetts, USA), anti-CDK4 (#12790, 1:1000, Cell Signaling Tech., Danvers, Massachusetts, USA), anti-CDK6 (#13331, 1:1000, Cell Signaling Tech., Danvers, Massachusetts, USA), anti-HBsAg (ab9193, 1:500, Abcam, Cambridge, UK), anti-GAPDH (ab8245, 1:8000, Abcam, Cambridge, UK), and anti-β-Tubulin (ab6046, 1:8000, Abcam, Cambridge, UK).

### Real-time quantitative PCR

From cells transfected with corresponding plasmids, total RNA was extracted using the Trelief™ RNAprep FastPure Tissue & Cell Kit (Tsingke Biotech, Beijing, China) according to the manufacturer's instructions. Complementary DNA synthesis reactions from the 1.5 μg RNA reverse transcription were carried out using the Transcriptor cDNA Synth. Kit 2 (Roche, Indianapolis, IN, USA). The quantitative PCR procedure utilized the PerfectStart™ Green qPCR SuperMix (TransGen Biotech, Beijing, China) with corresponding primers (Table 1) on Applied Biosystems 7500 Fast Real-Time System (Applied Biosystems, Waltham, MA, USA). The 2^–ΔΔCt^ method^16^ was applied to quantify relative expression of the target transcript, with GAPDH as the internal control.

**Table 1:** List of primers for quantitative PCR.

Primer	Sequence (5′–3′)
Akt-F	GTGCTGGAGGACAACGACT
Akt-R	GTGTAGGGTCCTTCTTGAGCA
mTOR-F	CCCAGCTGCTGGAACAAAAA
mTOR-R	GTCATGCCCACGTTCCTTAAC
CCND1-F	GAGAAGTTGTGCATCTACACTG
CCND1-R	AAATGAACTTCACATCTGTGGC
Rb1-F	GAACATCGAATCATGGAATCCCT
Rb1-R	AGAGGACAAGCAGATTCAAGGTGAT
CDK4-F	AATGTTGTACGGCTGATGGA
CDK4-R	AGAAACTGACGCATTAGATCCT
CDK6-F	ACTGGACCGGGCCTTTAG
CDK6-R	GAGAAGGTCTCTGTCCTC
GAPDH-F	ACCACAGTCCATGCCATCAC
GAPDH-R	TCCACCACCCTGTTGCTGTA

### Flow cytometry

Cell cycle distribution in Huh-7 cells after transfection was examined via flow cytometry using a Cell Cycle Assay Kit (Dojindo, Kumamoto, Japan) in accordance with the manufacturer's instructions. In brief, cells were collected 48 h post-transfection and fixed in pre-cooled (−20 °C) 70% ethanol at 4 °C for 2 h. Following ethanol removal via centrifugation (1000 *g*, 3 min) and two PBS washes of the cells, cell pellets were resuspended in staining solution supplemented with propidium iodide (PI) and RNase A. Cells were then incubated sequentially at 37 °C and 4 °C (30 min each) in the dark, followed by filtration through a 200-μm mesh to eliminate aggregates. Samples were detected by BD FACSCanto™ II flow cytometer (Becton, Dickinson and Company, New York, New Jersey, USA) using 488 nm excitation with PI emission signals > 620 nm. For each sample, data were acquired from 10,000 single cells, and the cell cycle was analyzed by ModFit LT 5.0 software (Verity Software House, Topsham, Maine, USA).

### Statistical analysis

The SPSS 21.0 software (SPSS Inc., Chicago, Illinois, USA) and GraphPad Prism 9.0 (GraphPad Software Inc., San Diego, California, USA) were utilized to conduct statistical analysis. The study employed the one-way analysis of variance (ANOVA) or unpaired two-tailed *t*-test for comparing the expression level of intracellular HBV surface proteins, the LHBs/SHBs ratio, and the protein and transcript levels of key molecules in the Akt/mTOR and cell cycle pathways among different groups. Two-way ANOVA was applied to assess the differences in cell proliferation levels and cell cycle distribution. Data from a minimum of three independent experiments are described as mean ± standard deviation. A two-tailed *P* value < 0.05 was considered statistically significant; the mean differences and 95% confidence intervals (CIs) were also estimated for the comparisons.

## Results

### Several high-frequency pre-S mutations in OBI significantly promote cell proliferation

**Figure 1:**
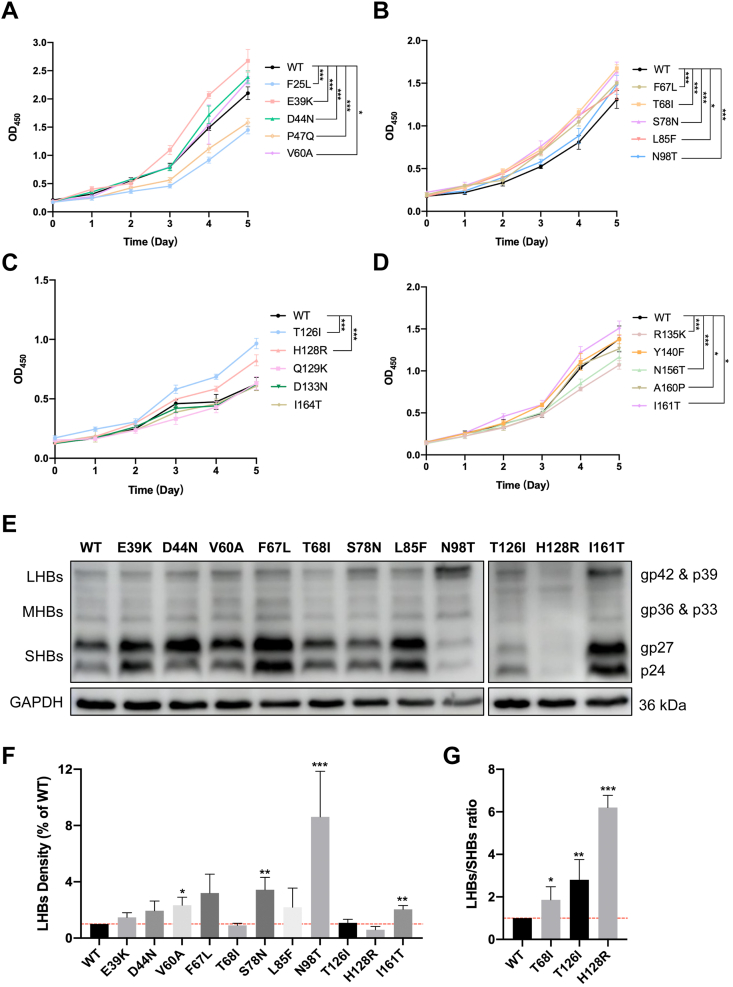
Several high-frequency pre-S mutations identified in occult HBV infection (OBI) significantly promote cell proliferation. Huh-7 cells were transfected with wild-type (WT) or 20 OBI-associated high-frequency pre-S mutant plasmids. Cells were harvested at 48 h and 72 h after transfection, and conducted cell proliferation assays and Western blotting analysis were conducted, respectively. **(A, B)** Cell proliferation assay results for 10 pre-S1 mutations (*n* = 3). **(C, D)** Cell proliferation assay results for 10 pre-S2 mutations (*n* = 3). **(E)** Representative Western blotting images of intracellular hepatitis B surface antigen (HBsAg) levels in cells transfected with 11 proliferation-promoting pre-S mutant plasmids. LHBs, MHBs, and SHBs denote the large, middle, and small hepatitis B surface proteins, respectively. **(F)** Absolute intracellular expression levels of LHBs, quantified by the densitometric values of LHBs bands (mutant groups are presented relative to WT, *n* = 3). **(G)** LHBs/SHBs ratio in cells transfected with pre-S T68I, T126I, and H128R plasmids, calculated from the densitometric values of SHBs and LHBs bands (*n* = 3). Detailed statistical results are provided in Table S1. ∗*P* < 0.05, ∗∗*P* < 0.01, and ∗∗∗*P* < 0.001.

To evaluate the impact of high-frequency OBI-related pre-S mutations on cell proliferation, cell proliferation analysis was conducted on the transfected cells. We found that several mutations in the pre-S1 region (E39K, D44N, V60A, F67L, T68I, S78N, L85F, and N98T) and in the pre-S2 region (T126I, H128R, and I161T) significantly enhanced proliferation of Huh-7 cells (Fig. 1A–D). Considering that these mutations reside within the HBsAg coding region, their effects on HBsAg expression were subsequently assessed by Western blotting analysis. The results showed that most of the proliferation-promoting mutations directly increased the intracellular absolute LHBs expression levels to varying degrees (Fig. 1E and F). Notably, although the T68I, T126I, and H128R mutations did not elevate the absolute levels of LHBs, they significantly increased the LHBs/SHBs ratio, reflecting a relative enhancement of LHBs expression (Fig. 1G). Detailed statistical results are provided in Table S1 (applicable to subsequent results as well). These results indicated that the proliferative capacity of these mutations may result from imbalanced expression of HBV surface proteins, especially the up-regulation of LHBs.

### Five OBI-associated high-frequency pre-S mutations activate the Akt/mTOR pathway

**Figure 2:**
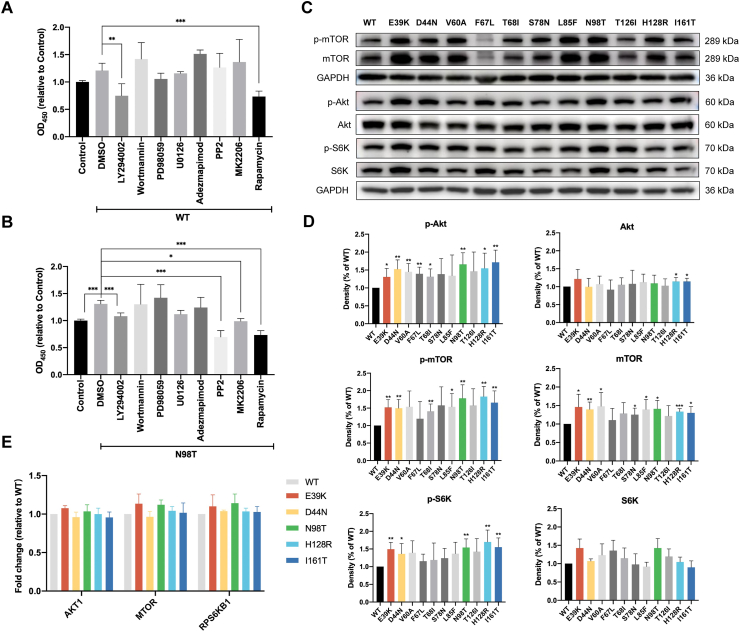
Five occult HBV infection (OBI)-associated high-frequency pre-S mutations activate the Akt/mTOR pathway. **(A, B)** Cell proliferation assays of Huh-7 cells transiently transfected with empty vector (Control), wild-type (WT), or N98T mutant plasmids following 24-h treatment with DMSO, LY294002 (50 μM), Wortmannin (10 nM), PD098059 (50 μM), U0126 (20 μM), Adezmapimod (10 μM), PP2 (10 μM), MK2206 (5 μM), or Rapamycin (10 nM) (*n* = 5). **(C)** Representative Western blotting images showing the expression of key molecules in the Akt/mTOR signaling pathway in Huh-7 cells transfected with pro-proliferative pre-S mutant plasmids. **(D)** Densitometric analysis of protein bands from panel C (*n* = 4). **(E)** Transcript levels of AKT1, MTOR, and RPS6KB1 (encoding Akt, mTOR, and S6K, respectively) in Huh-7 cells transfected with E39K, D44N, N98T, H128R, or I161T mutant plasmids (*n* = 3). Detailed statistical results are provided in Table S1. ∗*P* < 0.05, ∗∗*P* < 0.01, and ∗∗∗*P* < 0.001.

To explore the potential signaling underlying the pro-proliferative capacity of OBI-associated pre-S mutations, we selected the N98T mutation, which exhibited the most pronounced intracellular accumulation of LHBs, and conducted proliferation assays in Huh-7 cells treated with small-molecular inhibitors targeting various signaling pathways, including PI3K (LY294002 and Wortmannin), ERK (PD98059), MEK (U0126), p38/MAPK (Adezmapimod), Src (PP2), Akt (MK2206), and mTOR (Rapamycin). In WT plasmid-transfected cells, only LY294002 and Rapamycin significantly suppressed proliferation compared with DMSO controls. In contrast, in N98T-transfected cells, LY294002, PP2, MK2206, and Rapamycin all markedly suppressed cell proliferation relative to DMSO treatment. Given that Src and PI3K are upstream regulators of the Akt/mTOR pathway, it was likely that the proliferative effects induced by pre-S mutations are mediated through this signaling axis (Fig. 2A and B). Based on these findings, we employed Western blotting analysis to assess the effects of 11 OBI-associated pre-S mutations with proliferative capacity on the Akt/mTOR pathway. The results showed that among the 11 mutations, five mutations—E39K, D44N, N98T, H128R, and I161T—significantly enhanced the phosphorylation of Akt, mTOR, and the downstream effector S6 kinase (p-Akt, p-mTOR, and p-S6K; Fig. 2C and D), whereas the mRNA expression levels of the corresponding genes remained unchanged (Fig. 2E). These findings indicated that five OBI-associated pre-S mutations (E39K, D44N, N98T, H128R, and I161T) activated the Akt/mTOR signaling pathway. Therefore, we subsequently carried out a more detailed mechanistic investigation of these five mutations.

### Five OBI-associated high-frequency pre-S mutations up-regulate Cyclin D1 expression and promote G1/S cell cycle progression

**Figure 3:**
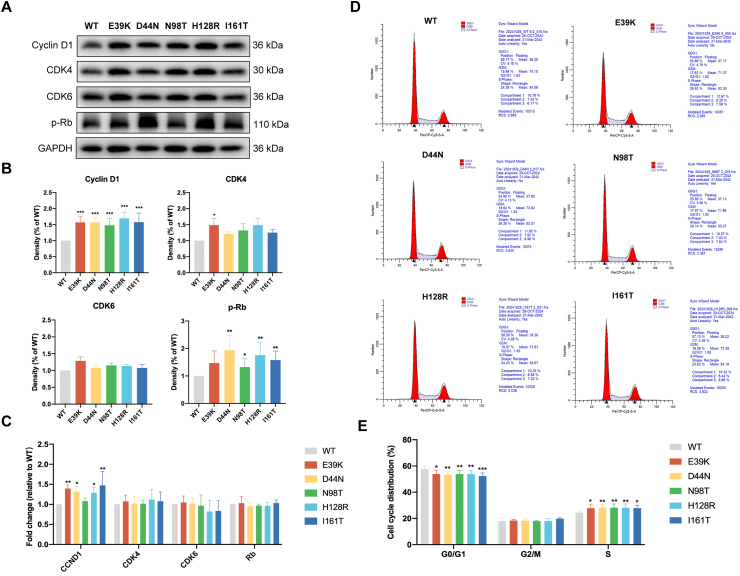
Five occult HBV infection (OBI)-associated high-frequency pre-S mutations up-regulate Cyclin D1 expression and promote G1/S cell cycle progression. Huh-7 cells were transfected with wild-type (WT), E39K, D44N, N98T, H128R, or I161T mutant plasmids. Cells were collected at 72 h after transfection for Western blotting analysis, and at 48 h for real-time quantitative PCR and flow cytometry. **(A)** Representative Western blotting images showing expression levels of Cyclin D1, Cyclin-dependent kinase (CDK) 4, CDK6, and phosphorylated retinoblastoma protein (p-Rb) in transfected Huh-7 cells. **(B)** Densitometric analysis of Western blot protein bands from panel A (*n* = 4). **(C)** Transcript levels of CCND1 (encoding Cyclin D1), CDK4, CDK6, and Rb in transfected Huh-7 cells (*n* = 3). **(D, E)** Results of cell cycle distribution in WT- or pre-S mutant-transfected Huh-7 cells detected by flow cytometry (*n* = 5). Detailed statistical results are provided in Table S1. ∗*P* < 0.05, ∗∗*P* < 0.01, and ∗∗∗*P* < 0.001.

To further clarify the mechanism by which the five OBI-associated pre-S mutations enhance cell proliferation through activation of the Akt/mTOR pathway, we examined Cyclin D1—a critical regulator of the cell cycle downstream of Akt/mTOR signaling—as well as other cell cycle-related proteins. The effects of these mutations on the expression of Cyclin D1, retinoblastoma protein (Rb), Cyclin-dependent kinase 4 (CDK4), and CDK6 were assessed by Western blotting and real-time quantitative PCR. All five mutations significantly up-regulated intracellular Cyclin D1 protein levels, accompanied by varying degrees of increased phosphorylation of Rb (p-Rb). A moderate up-regulation of CDK4 and CDK6 expression was also detected (Fig. 3A and B). At the transcriptional level, the mutations significantly elevated Cyclin D1 mRNA levels, although the increase induced by the N98T mutation did not reach statistical significance (Fig. 3C). Flow cytometry analysis further revealed that these mutations markedly reduced the percentage of cells in the G0/G1 phase, while concurrently increasing the percentage of S-phase cells, indicating enhanced G1/S cell cycle progression (Fig. 3D and E). These findings demonstrated that the OBI-associated mutations E39K, D44N, N98T, H128R, and I161T promote G1/S transition by up-regulating Cyclin D1 expression.

### The Akt/mTOR pathway serves as the critical mediator for cell cycle dysregulation and proliferation promotion induced by five OBI-associated pre-S mutations

**Figure 4:**
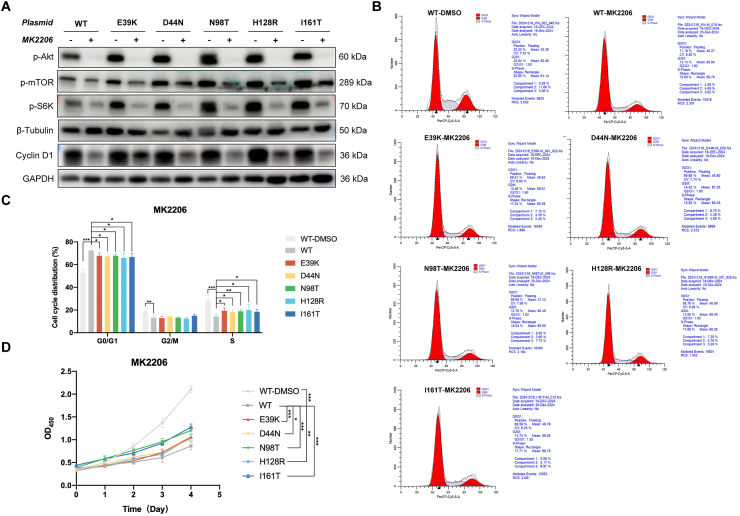
Influence of Akt inhibition on cell cycle progression and proliferation. Huh-7 cells transfected with wild-type (WT) or E39K, D44N, N98T, H128R, I161T mutant plasmids were treated with DMSO or Akt inhibitor (MK2206, 5 μM) for 24 h, followed by Western blotting, flow cytometry, and cell proliferation assays. **(A)** Representative Western blotting images of the levels of phosphorylated Akt, mTOR, S6K, and Cyclin D1 following MK2206 treatment. **(B)** Flow cytometric analysis of cell cycle distribution in transfected cells treated with MK2206. **(C)** Quantitative summary of cell cycle analysis from panel B (*n* = 5). **(D)** Cell proliferation analysis of transfected cells following MK2206 treatment (*n* = 3). Detailed statistical results are provided in Table S1. ∗*P* < 0.05, ∗∗*P* < 0.01, and ∗∗∗*P* < 0.001.

**Figure 5:**
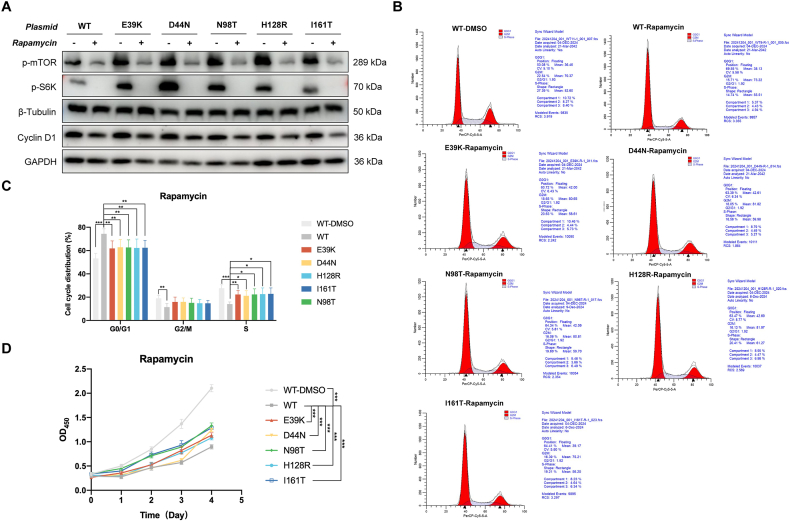
Influence of mTOR inhibition on cell cycle progression and proliferation. Huh-7 cells transfected with wild-type (WT) or E39K, D44N, N98T, H128R, I161T mutant plasmids were treated with DMSO or the mTOR inhibitor Rapamycin (60 nM) for 24 h, followed by Western blotting, flow cytometry, and cell proliferation assays. **(A)** Representative Western blotting images showing the levels of phosphorylated mTOR, S6K, and Cyclin D1 following Rapamycin treatment. **(B)** Flow cytometric analysis of cell cycle distribution in transfected cells treated with Rapamycin. **(C)** Quantitative summary of cell cycle analysis from panel B (*n* = 5). **(D)** Cell proliferation analysis of transfected cells following Rapamycin treatment (*n* = 3). Detailed statistical results are provided in Table S1. ∗*P* < 0.05, ∗∗*P* < 0.01, and ∗∗∗*P* < 0.001.

To confirm the role of the Akt/mTOR pathway in mediating cell cycle dysregulation and enhanced proliferation induced by the five OBI-associated pre-S mutations, transfected Huh-7 cells were treated with small-molecule inhibitors targeting Akt (MK2206) or mTOR (Rapamycin). Western blotting analysis validated effective suppression of Akt/mTOR signaling following inhibitor treatment, accompanied by a marked down-regulation of Cyclin D1 level (Figure 4, Figure 5A). Treatment with MK2206 or Rapamycin elevated the percentage of cells in the G0/G1 phase, while reducing the S and G2/M phases populations in both WT- and mutant-transfected cells compared with DMSO treatment, indicating inhibitor-induced cell cycle arrest. Notably, the E39K, D44N, N98T, H128R, and I161T mutations partially mitigated the inhibitor-induced G0/G1 phase accumulation and S-phase reduction relative to WT cells (Figure 4, Figure 5B, C). Consistent with these results, cell proliferation assays demonstrated reduced anti-proliferative effects of MK2206 and Rapamycin in both WT and mutant groups compared with DMSO controls, while the five mutations partially rescued the proliferation suppression observed in WT cells (Figure 4, Figure 5D). Collectively, these findings demonstrated that the Akt/mTOR signaling constitutes a key mechanism by which these pre-S mutations promote G1/S cell cycle transition and enhance cellular proliferation.

### LHBs mediated Akt/mTOR/Cyclin D1 pathway activation and proliferation induced by OBI-associated pre-S mutations

**Figure 6:**
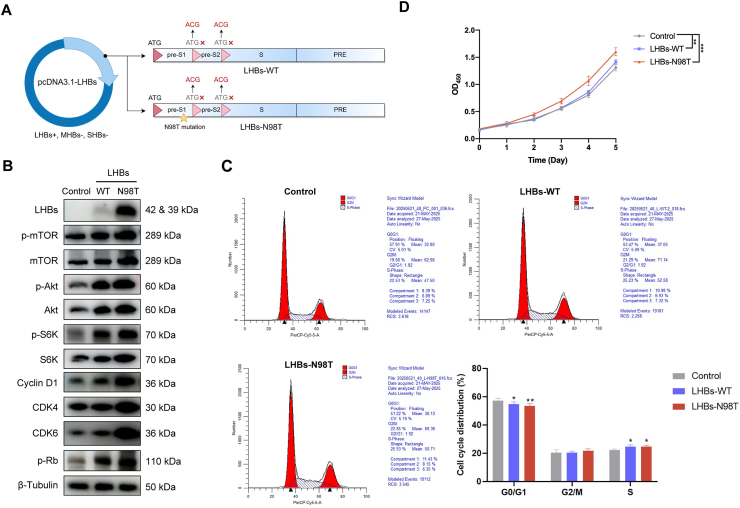
Effects of large hepatitis B surface protein (LHBs) on Akt/mTOR/Cyclin D1 pathway activation, cell cycle progression, and cellular proliferation. **(A)** Schematic diagram of the LHBs expression plasmid. HBV pre-S/S and posttranscriptional regulatory element (PRE) sequences were cloned into the pcDNA 3.1 vector. Site-directed mutagenesis was employed to construct plasmids encoding either wild-type LHBs (LHBs-WT) or N98T-mutant LHBs (LHBs-N98T) exclusively. **(B)** Western blotting results of critical components of the Akt/mTOR/Cyclin D1 pathway in Huh-7 cells expressing LHBs-WT or LHBs-N98T. **(C)** Results of cell cycle distribution following LHBs expression detected by flow cytometry (*n* = 4). **(D)** Proliferation assay results of Huh-7 cells expressing LHBs-WT or LHBs-N98T (*n* = 3). Detailed statistical results are provided in Table S1. ∗*P* < 0.05, ∗∗*P* < 0.01, and ∗∗∗*P* < 0.001.

Given the up-regulation of intracellular LHBs expression by most of these five OBI-associated pre-S mutations (Fig. 1E–G), we next constructed plasmids only expressing either WT LHBs or N98T-mutated LHBs (exhibiting the highest LHBs accumulation; Fig. 6A), to evaluate the regulatory role of LHBs in Akt/mTOR pathway activation, the expression level of Cyclin D1 and associated cell cycle regulators, dynamic cell cycle progression, and proliferation. Western blotting analysis revealed that both LHBs-WT and LHBs-N98T groups displayed significantly elevated phosphorylation of mTOR, Akt, and S6K compared with the pcDNA 3.1 vector control, with a positive correlation between protein phosphorylation and LHBs levels. The expression of Cyclin D1, p-Rb, CDK4, and CDK6 was also elevated in the LHBs-expressing groups (Fig. 6B). Additionally, LHBs expression accelerated the G1/S phase transition and enhanced proliferative capacity, particularly in cells expressing the N98T-mutant LHBs, which showed more pronounced pro-proliferative effects than WT LHBs (Fig. 6C and D). Collectively, these findings established LHBs as a key mediator through which OBI-associated pre-S mutations activate the Akt/mTOR/Cyclin D1 signaling cascade, promote G1/S cell cycle progression, and drive aberrant cell proliferation.

## Discussion

**Figure 7:**
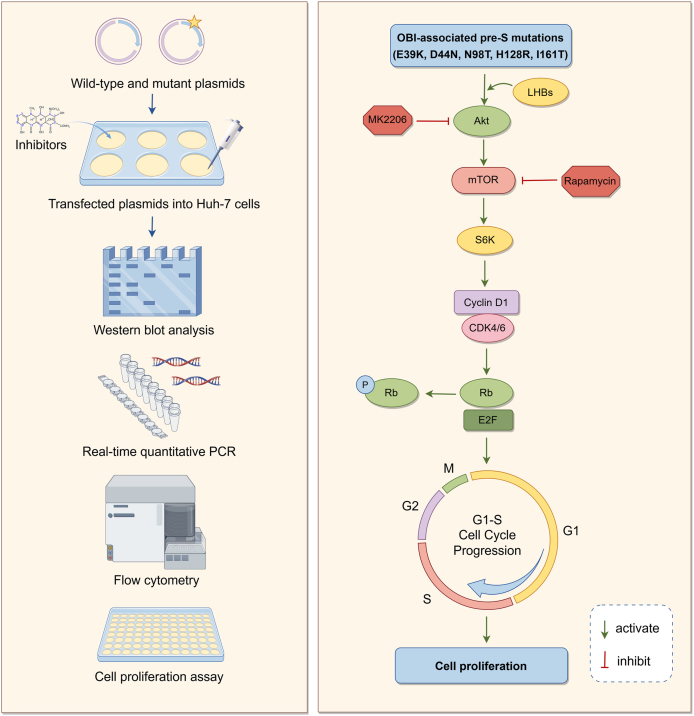
Occult hepatitis B virus pre-S mutations lead to cell cycle dysregulation and aberrant proliferation through activation of the Akt/mTOR/Cyclin D1 signaling pathway. Employing an experimental approach involving cell transfection, Western blotting analysis, real-time quantitative PCR, flow cytometry, cell proliferation analysis, and inhibitor treatment (left panel), this study demonstrated that occult HBV infection (OBI)-associated pre-S mutations (E39K, D44N, N98T, H128R, and I161T) activate Akt/mTOR signaling pathway, up-regulate Cyclin D1 expression, promote retinoblastoma protein (Rb) phosphorylation, drive G1/S phase transition in the cell cycle, and ultimately enhance cell proliferation. Large hepatitis B surface protein (LHBs) likely plays a critical role in mediating the process (right panel). mTOR, mammalian target of rapamycin; S6K, S6 kinase; CDK, cyclin-dependent kinase.

Among patients with HBV-associated HCC, approximately 20% have been identified as OBI-related,^17^ highlighting a strong association between OBI and hepatocarcinogenesis. However, the precise molecular pathways underlying this relationship remain largely unclear. Increasing evidence points to a close association between pre-S deletion mutations and the development and progression of HCC, and they are also linked to poor prognosis and influence therapeutic decision-making in HCC patients.^18^ However, studies about the pre-S point mutations remain very limited. In the present study, we paid attention to the high-frequency pre-S point mutations identified in OBI and demonstrated that the E39K, D44N, N98T, H128R, and I161T mutations promote hepatocyte proliferation by activating the Akt/mTOR signaling pathway, up-regulating Cyclin D1 expression, and inducing G1/S cell cycle progression. LHBs likely serve as the pivotal mediators in this process (Fig. 7). To our knowledge, this study represents the first attempt to explore the molecular mechanisms by which OBI-related high-frequency pre-S point mutations promote cell proliferation and hepatocarcinogenesis.

Using cell proliferation assays, we first identified that 11 high-frequency pre-S mutations found in OBI blood donors, such as E39K, D44N, and V60A, could promote the proliferation of Huh-7 cells (Fig. 1). These findings suggest that pre-S point mutations may possess oncogenic potential and contribute to HCC development, warranting further mechanistic investigation. By employing small-molecule inhibitors targeting multiple signaling pathways, we identified that the Akt/mTOR axis plays a critical regulatory role in the proliferative effects induced by OBI-related pre-S mutations. The Akt/mTOR signaling cascade is a critical regulator of cellular growth, survival, and metabolic processes, and its dysregulation is commonly observed in HCC. As a key downstream effector, mTOR operates through two complexes (mTORC1 and mTORC2), with most research focusing on mTORC1. Through phosphorylation of its specific substrates S6K, mTORC1 promotes protein synthesis, thus regulating cell growth and proliferation.^19^^,^^20^ Studies indicated that HBV X protein, surface protein, and pre-S deletion mutations could activate the Akt/mTOR pathway to promote hepatocarcinogenesis.^21^, ^22^, ^23^ In the present study, the OBI-related high-frequency pre-S mutations E39K, D44N, N98T, H128R, and I161T were found to significantly activate the Akt/mTOR signaling, with the elevated phosphorylation of S6K indicating enhanced activity of the mTORC1 complex (Fig. 2). In contrast, other mutations (V60A, F67L, T68I, S78N, L85F, and T126I) exhibited no such effect, suggesting that distinct pre-S mutations may differentially regulate downstream signaling pathways. Further mechanistic studies are warranted to fully characterize the diverse oncogenic properties of these mutations. In addition, recent studies have begun to differentiate the functions of mTORC1 and mTORC2 and explore their distinct roles in cancer,^24^ providing a direction for future research on the specific regulation of mTOR complexes by pre-S mutations.

Emerging evidence suggests that Cyclin D1 functions as a critical downstream effector of the Akt/mTOR axis in regulating cell growth and proliferation. Activation of mTOR enhances Cyclin D1 synthesis, which subsequently forms complexes with CDK4/6, resulting in phosphorylation of Rb and facilitating G1-to-S phase transition.^25^^,^^26^ Furthermore, aberrant Cyclin D1 overexpression has been shown to facilitate tumor progression and correlates with poor prognosis in HCC.^27^^,^^28^ In this study, we provide the first evidence that five OBI-associated pre-S point mutations—E39K, D44N, N98T, H128R, and I161T—markedly up-regulated Cyclin D1 expression, increased phosphorylation of Rb, and promoted G1/S phase progression in Huh-7 cells (Fig. 3). Furthermore, rescue experiments using Akt and mTOR inhibitors validated that the proliferative advantage conferred by these mutations is critically dependent on the Akt/mTOR/Cyclin D1 signaling axis (Figure 4, Figure 5). Specifically, the mTOR inhibitor Rapamycin specifically targets mTORC1 rather than mTORC2, thereby allowing validation of the effects of those pre-S mutations on mTORC1 activity. These findings offer mechanistic insights into the tumorigenic potential of OBI-associated pre-S mutations and may inform the development of targeted therapies for HBV-related HCC.

We further explored how OBI-associated pre-S point mutations activate the Akt/mTOR/Cyclin D1 axis and promote cellular proliferation. We examined the impacts of these mutations on HBsAg expression and found that most proliferative mutations up-regulated intracellular absolute LHBs expression levels, while certain mutations, such as T68I, T126I, and H128R, did not increase absolute LHBs expression but significantly elevated the LHBs/SHBs ratio, resulting in a relative overexpression of LHBs (Fig. 1E–G). These findings suggest that different pre-S mutations may differentially alter surface protein composition. Using plasmids expressing either WT or N98T-mutated LHBs, we demonstrated that LHBs overexpression was sufficient to activate the Akt/mTOR signaling cascade, up-regulate Cyclin D1 and related cell cycle regulators, enhance G1-to-S phase transition, and promote cellular proliferation (Fig. 6). Notably, the effects were more pronounced in cells expressing the N98T-mutated LHBs. These results identify LHBs as the central mediator linking pre-S mutations to oncogenic signaling activation. Previous studies have implicated LHBs, among the three HBV surface proteins, as the one most strongly associated with hepatocarcinogenesis.^29^^,^^30^ The oncogenic effect of LHBs overexpression—particularly in the context of pre-S deletions—has also been documented,^31^ further supporting our findings. In addition to contributing to hepatocarcinogenesis, LHBs are also closely associated with the prognosis of the disease. Studies have shown that the higher LHBs levels in serum or liver are strongly correlated with advanced stage and poor clinical outcomes, including decreased overall and progression-free survival.^29^ Future studies should further investigate the molecular mechanisms by which LHBs promote the development and progression of HCC and evaluate their potential as novel biomarkers for the disease.

This study primarily focused on the impact of OBI-associated pre-S point mutations on cell proliferation and hepatocarcinogenesis, without involving other aspects of HCC development, representing a limitation. In fact, pre-S mutations have significant importance in the occurrence, progression, prognosis assessment, and treatment of HCC. Pre-S mutations are closely related to microvascular invasion in liver cancer, tumor growth, and abnormal liver function in patients, and serve as trustworthy biomarkers for HCC prognosis prediction.^32^ Expression of pre-S deletion variants shows a significant correlation with a higher likelihood of recurrence of HCC following curative resection, despite the administration of antiviral therapy before surgery.^33^, ^34^, ^35^, ^36^ Additionally, pre-S deletions have been reported to confer resistance to 5-fluorouracil and antiviral therapies, thereby impairing treatment efficacy.^37^^,^^38^ Therefore, monitoring pre-S mutations in HBV-infected individuals is essential across multiple stages of HBV-related HCC pathogenesis.

In conclusion, this study systematically investigated high-frequency OBI-associated pre-S point mutations and identified 11 mutations that significantly enhance hepatocyte proliferation. Among them, five mutations (E39K, D44N, N98T, H128R, and I161T) were shown to activate the Akt/mTOR/Cyclin D1 signaling axis, resulting in dysregulated cell cycle progression and abnormal proliferation. These findings offer novel mechanistic understandings regarding the oncogenic potential of pre-S point mutations in hepatocarcinogenesis and may contribute to the identification of high-risk individuals within OBI populations as well as the development of therapeutic approaches for treating HBV-associated HCC. Future studies integrating basic and clinical research are warranted to elucidate the role and molecular mechanisms of pre-S mutations across various stages of HCC development.

## CRediT authorship contribution statement

**Huizhen Sun:** Writing – review & editing, Writing – original draft, Visualization, Validation, Resources, Project administration, Methodology, Investigation, Formal analysis, Data curation, Conceptualization. **Ling Mei:** Writing – review & editing, Writing – original draft, Visualization, Validation, Methodology, Investigation, Formal analysis, Data curation, Conceptualization. **Shi Song:** Methodology, Investigation, Formal analysis, Data curation. **Qian Su:** Methodology, Formal analysis, Conceptualization. **Ying Yan:** Investigation, Formal analysis, Conceptualization. **Huimin Ji:** Methodology, Formal analysis, Conceptualization. **Jie Ma:** Resources, Investigation, Formal analysis, Conceptualization. **Le Chang:** Writing – review & editing, Validation, Resources, Project administration, Investigation, Funding acquisition, Formal analysis, Data curation, Conceptualization. **Lunan Wang:** Writing – review & editing, Visualization, Validation, Resources, Project administration, Methodology, Investigation, Funding acquisition, Formal analysis, Data curation, Conceptualization.

## Funding

This work was supported by the grants from the 10.13039/501100001809National Natural Science Foundation of China (No. 82202612) and the 10.13039/501100004826Beijing Natural Science Foundation (China) (No. 7232142).

## Conflict of interests

All authors have no conflict of interests to disclose.
