# Supplementary material for: Potential oncogenic role of occult hepatitis B virus pre-S mutations: Activation of Akt/mTOR/Cyclin D1 signaling drives cell cycle dysregulation and proliferation in hepatocellular carcinogenesis
Source: Genes Dis. 2025 Nov 6;13(5):101919. doi: 10.1016/j.gendis.2025.101919 (PMC13285653; doi:10.1016/j.gendis.2025.101919)
Supplement: Multimedia component 1 [file mmc1.docx]

**Supplementary Table S1.** Summary of statistical analysis.

| Comparison  (Group A vs Group B) | Mean difference (Group B-Group A) ^a^ | 95% CI of difference ^b^ | *P* value | n per group ^c^ | Figures ^d^ |
| --- | --- | --- | --- | --- | --- |
| WT vs F25L | -0.65 | -0.83 to -0.47 | <0.001 | 3 | 1A |
| WT vs E39K | 0.57 | 0.39 to 0.76 | <0.001 | 3 | 1A |
| WT vs D44N | 0.29 | 0.11 to 0.47 | <0.001 | 3 | 1A |
| WT vs P47Q | -0.52 | -0.70 to -0.34 | <0.001 | 3 | 1A |
| WT vs V60A | 0.27 | 0.14 to 0.41 | 0.012 | 3 | 1A |
| WT vs F67L | 0.20 | 0.09 to 0.30 | <0.001 | 3 | 1B |
| WT vs T68I | 0.36 | 0.26 to 0.47 | <0.001 | 3 | 1B |
| WT vs S78N | 0.33 | 0.22 to 0.44 | <0.001 | 3 | 1B |
| WT vs L85F | 0.15 | 0.08 to 0.22 | 0.045 | 3 | 1B |
| WT vs N98T | 0.27 | 0.06 to 0.48 | <0.001 | 3 | 1B |
| WT vs T126I | 0.34 | 0.27 to 0.41 | <0.001 | 3 | 1C |
| WT vs H128R | 0.20 | 0.13 to 0.26 | <0.001 | 3 | 1C |
| WT vs R135K | -0.31 | -0.42 to -0.20 | <0.001 | 3 | 1D |
| WT vs N156T | -0.22 | -0.32 to -0.11 | <0.001 | 3 | 1D |
| WT vs A160P | -0.12 | -0.22 to -0.01 | 0.026 | 3 | 1D |
| WT vs I161T | 0.14 | 0.05 to 0.23 | 0.014 | 3 | 1D |
| WT vs V60A | 1.34 | 0.43 to 2.24 | 0.015 | 3 | 1F |
| WT vs S78N | 2.43 | 1.02 to 3.84 | 0.008 | 3 | 1F |
| WT vs N98T | 7.61 | 4.73 to 10.50 | <0.001 | 3 | 1F |
| WT vs I161T | 1.14 | 0.61 to 1.67 | 0.003 | 3 | 1F |
| WT vs T68I | 0.86 | 0.22 to 1.50 | 0.015 | 3 | 1G |
| WT vs T126I | 1.80 | 0.81 to 2.79 | 0.004 | 3 | 1G |
| WT vs H128R | 5.20 | 4.64 to 5.76 | <0.001 | 3 | 1G |
| DMSO vs LY294002 | -0.46 | -0.82 to -0.10 | 0.008 | 5 | 2A |
| DMSO vs Rapamycin | -0.51 | -0.56 to -0.46 | <0.001 | 5 | 2A |
| DMSO vs Control | -0.31 | -0.38 to -0.24 | <0.001 | 5 | 2B |
| DMSO vs LY294002 | -0.23 | -0.32 to -0.14 | <0.001 | 5 | 2B |
| DMSO vs PP2 | -0.61 | -0.91 to -0.32 | <0.001 | 5 | 2B |
| DMSO vs MK2206 | -0.32 | -0.61 to -0.03 | 0.027 | 5 | 2B |
| DMSO vs Rapamycin | -0.57 | -0.87 to -0.28 | 0.006 | 5 | 2B |
| WT vs E39K | 0.31 | 0.11 to 0.50 | 0.041 | 4 | 2D (p-Akt) |
| WT vs D44N | 0.53 | 0.21 to 0.84 | 0.006 | 4 | 2D (p-Akt) |
| WT vs V60A | 0.45 | 0.17 to 0.73 | 0.007 | 4 | 2D (p-Akt) |
| WT vs F67L | 0.40 | 0.17 to 0.62 | 0.005 | 4 | 2D (p-Akt) |
| WT vs T68I | 0.34 | 0.10 to 0.57 | 0.026 | 4 | 2D (p-Akt) |
| WT vs N98T | 0.66 | 0.26 to 1.06 | 0.008 | 4 | 2D (p-Akt) |
| WT vs H128R | 0.55 | 0.13 to 1.07 | 0.041 | 4 | 2D (p-Akt) |
| WT vs I161T | 0.72 | 0.29 to 1.14 | 0.007 | 4 | 2D (p-Akt) |
| WT vs H128R | 0.18 | 0.09 to 0.28 | 0.041 | 4 | 2D (Akt) |
| WT vs I161T | 0.15 | 0.05 to 0.25 | 0.02 | 4 | 2D (Akt) |
| WT vs E39K | 0.52 | 0.24 to 0.80 | 0.004 | 4 | 2D (p-mTOR) |
| WT vs D44N | 0.50 | 0.19 to 0.80 | 0.007 | 4 | 2D (p-mTOR) |
| WT vs T68I | 0.41 | 0.16 to 0.66 | 0.007 | 4 | 2D (p-mTOR) |
| WT vs L85F | 0.54 | 0.10 to 0.99 | 0.030 | 4 | 2D (p-mTOR) |
| WT vs N98T | 0.78 | 0.31 to 1.25 | 0.006 | 4 | 2D (p-mTOR) |
| WT vs H128R | 0.83 | 0.47 to 1.18 | 0.002 | 4 | 2D (p-mTOR) |
| WT vs I161T | 0.66 | 0.24 to 1.07 | 0.009 | 4 | 2D (p-mTOR) |
| WT vs E39K | 0.48 | 0.09 to 0.88 | 0.039 | 4 | 2D (mTOR) |
| WT vs D44N | 0.40 | 0.15 to 0.64 | 0.009 | 4 | 2D (mTOR) |
| WT vs V60A | 0.50 | 0.05 to 0.94 | 0.045 | 4 | 2D (mTOR) |
| WT vs S78N | 0.26 | 0.05 to 0.46 | 0.026 | 4 | 2D (mTOR) |
| WT vs L85F | 0.40 | 0.06 to 0.72 | 0.027 | 4 | 2D (mTOR) |
| WT vs N98T | 0.41 | 0.13 to 0.69 | 0.001 | 4 | 2D (mTOR) |
| WT vs H128R | 0.34 | 0.24 to 0.43 | <0.001 | 4 | 2D (mTOR) |
| WT vs I161T | 0.30 | 0.09 to 0.51 | 0.013 | 4 | 2D (mTOR) |
| WT vs E39K | 0.50 | 0.26 to 0.73 | 0.002 | 4 | 2D (p-S6K) |
| WT vs D44N | 0.40 | 0.08 to 0.72 | 0.048 | 4 | 2D (p-S6K) |
| WT vs N98T | 0.54 | 0.24 to 0.84 | 0.006 | 4 | 2D (p-S6K) |
| WT vs H128R | 0.70 | 0.28 to 1.12 | 0.008 | 4 | 2D (p-S6K) |
| WT vs I161T | 0.56 | 0.24 to 0.87 | 0.005 | 4 | 2D (p-S6K) |
| WT vs E39K | 0.57 | 0.27 to 0.87 | <0.001 | 4 | 3B (Cyclin D1) |
| WT vs D44N | 0.57 | 0.23 to 0.91 | <0.001 | 4 | 3B (Cyclin D1) |
| WT vs N98T | 0.48 | 0.18 to 0.78 | <0.001 | 4 | 3B (Cyclin D1) |
| WT vs H128R | 0.70 | 0.39 to 1.00 | <0.001 | 4 | 3B (Cyclin D1) |
| WT vs I161T | 0.58 | 0.28 to 0.88 | <0.001 | 4 | 3B (Cyclin D1) |
| WT vs E39K | 0.49 | 0.11 to 0.86 | 0.012 | 4 | 3B (CDK4) |
| WT vs D44N | 0.94 | 0.38 to 1.49 | 0.004 | 4 | 3B (p-Rb) |
| WT vs N98T | 0.39 | 0.14 to 0.63 | 0.043 | 4 | 3B (p-Rb) |
| WT vs H128R | 0.76 | 0.26 to 1.25 | 0.008 | 4 | 3B (p-Rb) |
| WT vs I161T | 0.58 | 0.24 to 0.92 | 0.004 | 4 | 3B (p-Rb) |
| WT vs E39K | 0.40 | 0.11 to 0.68 | 0.003 | 3 | 3C |
| WT vs D44N | 0.35 | 0.08 to 0.63 | 0.044 | 3 | 3C |
| WT vs H128R | 0.32 | 0.07 to 0.57 | 0.048 | 3 | 3C |
| WT vs I161T | 0.47 | 0.16 to 0.78 | 0.001 | 3 | 3C |
| WT vs E39K | -3.72 | -6.79 to -0.65 | 0.011 | 5 | 3E (G0/G1) |
| WT vs D44N | -4.22 | -7.29 to -1.14 | 0.003 | 5 | 3E (G0/G1) |
| WT vs N98T | -3.69 | -6.54 to -0.85 | 0.006 | 5 | 3E (G0/G1) |
| WT vs H128R | -3.74 | -6.41 to -0.99 | 0.005 | 5 | 3E (G0/G1) |
| WT vs I161T | -5.21 | -8.29 to -2.14 | <0.001 | 5 | 3E (G0/G1) |
| WT vs E39K | 3.70 | 0.99 to 6.41 | 0.028 | 5 | 3E (S) |
| WT vs D44N | 3.94 | 1.01 to 6.87 | 0.007 | 5 | 3E (S) |
| WT vs N98T | 3.79 | 1.04 to 6.53 | 0.006 | 5 | 3E (S) |
| WT vs H128R | 3.75 | 1.00 to 6.49 | 0.006 | 5 | 3E (S) |
| WT vs I161T | 3.72 | 0.90 to 6.54 | 0.022 | 5 | 3E (S) |
| WT vs WT-DMSO | -19.85 | -24.48 to -15.22 | <0.001 | 5 | 4C (G0/G1) |
| WT vs E39K | -5.39 | -9.41 to -1.37 | 0.038 | 5 | 4C (G0/G1) |
| WT vs D44N | -5.61 | -9.31 to -1.90 | 0.024 | 5 | 4C (G0/G1) |
| WT vs N98T | -5.29 | -9.57 to -1.00 | 0.048 | 5 | 4C (G0/G1) |
| WT vs H128R | -6.66 | -11.48 to -1.83 | 0.039 | 5 | 4C (G0/G1) |
| WT vs I161T | -6.30 | -11.37 to -1.22 | 0.044 | 5 | 4C (G0/G1) |
| WT vs WT-DMSO | 6.03 | 1.40 to 10.66 | 0.005 | 5 | 4C (G2/M) |
| WT vs WT-DMSO | 13.82 | 9.19 to 18.45 | <0.001 | 5 | 4C (S) |
| WT vs E39K | 4.88 | 1.25 to 8.51 | 0.017 | 5 | 4C (S) |
| WT vs D44N | 3.91 | 0.75 to 7.07 | 0.041 | 5 | 4C (S) |
| WT vs N98T | 4.47 | 1.55 to 7.39 | 0.009 | 5 | 4C (S) |
| WT vs H128R | 6.08 | 1.01 to 11.15 | 0.026 | 5 | 4C (S) |
| WT vs I161T | 4.27 | 1.10 to 7.45 | 0.029 | 5 | 4C (S) |
| WT vs WT-DMSO | 1.24 | 1.11 to 1.38 | <0.001 | 3 | 4D |
| WT vs E39K | 0.21 | 0.10 to 0.32 | <0.001 | 3 | 4D |
| WT vs D44N | 0.17 | 0.04 to 0.29 | 0.021 | 3 | 4D |
| WT vs N98T | 0.34 | 0.21 to 0.46 | <0.001 | 3 | 4D |
| WT vs H128R | 0.18 | 0.06 to 0.30 | 0.002 | 3 | 4D |
| WT vs I161T | 0.40 | 0.28 to 0.52 | <0.001 | 3 | 4D |
| WT vs WT-DMSO | -21.05 | -29.06 to -13.04 | <0.001 | 5 | 5C (G0/G1) |
| WT vs E39K | -12.57 | -21.69 to -3.45 | 0.003 | 5 | 5C (G0/G1) |
| WT vs D44N | -11.50 | -20.62 to -2.38 | 0.008 | 5 | 5C (G0/G1) |
| WT vs N98T | -11.66 | -20.78 to -2.54 | 0.007 | 5 | 5C (G0/G1) |
| WT vs H128R | -12.14 | -21.28 to -2.99 | 0.005 | 5 | 5C (G0/G1) |
| WT vs I161T | -11.91 | -21.03 to -2.79 | 0.006 | 5 | 5C (G0/G1) |
| WT vs WT-DMSO | 7.48 | 3.11 to 11.85 | 0.005 | 5 | 5C (G2/M) |
| WT vs WT-DMSO | 13.57 | 5.56 to 21.58 | <0.001 | 5 | 5C (S) |
| WT vs E39K | 8.19 | 3.19 to 13.20 | 0.008 | 5 | 5C (S) |
| WT vs D44N | 7.08 | 1.00 to 13.15 | 0.030 | 5 | 5C (S) |
| WT vs N98T | 7.87 | 1.23 to 14.52 | 0.038 | 5 | 5C (S) |
| WT vs H128R | 8.52 | 1.38 to 15.66 | 0.028 | 5 | 5C (S) |
| WT vs I161T | 8.69 | 1.78 to 15.61 | 0.023 | 5 | 5C (S) |
| WT vs WT-DMSO | 1.21 | 1.11 to 1.31 | <0.001 | 3 | 5D |
| WT vs E39K | 0.24 | 0.14 to 0.33 | <0.001 | 3 | 5D |
| WT vs D44N | 0.35 | 0.25 to 0.45 | <0.001 | 3 | 5D |
| WT vs N98T | 0.44 | 0.34 to 0.53 | <0.001 | 3 | 5D |
| WT vs H128R | 0.19 | 0.09 to 0.28 | <0.001 | 3 | 5D |
| WT vs I161T | 0.39 | 0.30 to 0.49 | <0.001 | 3 | 5D |
| Control vs LHBs-WT | -2.29 | -4.49 to -0.08 | 0.048 | 4 | 6C (G0/G1) |
| Control vs LHBs-N98T | -3.60 | -5.75 to -1.45 | 0.001 | 4 | 6C (G0/G1) |
| Control vs LHBs-WT | 2.81 | -4.63 to -1.00 | 0.040 | 4 | 6C (S) |
| Control vs LHBs-N98T | 2.62 | -4.37 to -0.87 | 0.042 | 4 | 6C (S) |
| Control vs LHBs-WT | 0.20 | 0.11 to 0.29 | 0.009 | 3 | 6D |
| Control vs LHBs-N98T | 0.29 | 0.21 to 0.37 | <0.001 | 3 | 6D |

a, The mean difference was calculated as the value of group B minus that of group A, with the units consistent with those presented in the corresponding figure.

b, CI: confidence intervals.

c, n per group: the number of independent biological replicates.

d, Exact values are provided here for statistical transparency; summary statistics are displayed in the corresponding Figures.
